# Clients’ satisfaction with HIV care and treatment centres in Dar es Salaam, Tanzania: A cross-sectional study

**DOI:** 10.1371/journal.pone.0247421

**Published:** 2021-02-22

**Authors:** Salome E. Buluba, Neema E. Mawi, Edith A. M. Tarimo

**Affiliations:** 1 Department of Clinical Nursing, School of Nursing, Muhimbili University of Health and Allied Sciences, Dar es Salaam, Tanzania; 2 Department of Nursing Management, School of Nursing, Muhimbili University of Health and Allied Sciences, Dar es Salaam, Tanzania; Freelance Consultant, MYANMAR

## Abstract

**Background:**

HIV is a major global public health challenge, claiming the lives of over 32 million people so far. The satisfaction of HIV-affected clients with the quality of their HIV services at treatment centres is crucial for quality improvement. This article assesses clients’ satisfaction with different aspects of the overall care experience and seeks to determine if the type of health facility ownership is a predictor of satisfaction.

**Methods:**

A cross-sectional study involving 430 respondents was conducted between September and October 2019. Purposeful and convenient sampling techniques were used to select health facilities and potential respondents, respectively. A pre-tested, interviewer-administered questionnaire was used to collect data. Binary logistic regression was used to assess the association between type of health facility and clients’ satisfaction based on the six assessed aspects of care, and p˂0.05 was considered statistically significant.

**Results:**

The general clients’ satisfaction with HIV/AIDS services at care and treatment centres was 92.3%. Respondents from public health facilities were most satisfied with privacy and confidentiality (100%), physical environment (100%), counseling (99.5%) and drug availability (99.5%); respondents from private health facilities were most satisfied with the time spent in the facility (95.9%); while respondents from faith-based health facilities were most satisfied with staff-patient communication (99.2%). However, after adjusting for confounders, only one aspect of care, that of “time spent in the facility,” showed significant association with the type of health facility.

**Conclusion:**

Generally, clients’ satisfaction with HIV/AIDS services at care and treatment centres in the Ubungo District, Dar es Salaam was high. This finding should encourage health care providers to maintain high-quality services to sustain clients’ satisfaction.

## Introduction

HIV continues to be a major global public health challenge, with a death toll of over 32 million people so far [1]. Globally in 2018, the total number of people living with HIV was estimated to be 37.9 million, out of which 1.7 million were newly infected. An additional 770,000 died due to HIV-related causes [1]. The disease burden continues to be substantial among Sub-Saharan countries, which have 69 percent of all HIV/AIDS cases around the globe [2]. In Sub-Saharan Africa, Tanzania has an HIV prevalence of 4.6% in the general population, with up to 72,000 new HIV infections and 24,000 AIDS-related deaths in 2018 [3].

The Ministry of Health of Tanzania, in collaboration with its development and implementing partners, has been striving to reduce the burden of this disease [4]. The focus of the national response is mainly on the prevention of HIV transmission, as well as the provision of care, treatment, and support to people living with HIV (PLHIV). Examples of prevention interventions include HIV testing services, antiretroviral therapy (ART), Prevention of Mother to Child Transmission (PMTCT), and voluntary male circumcision [5,6]. These efforts have resulted in a steady decrease of new HIV infections, from over 200,000 estimated annually in the early 1990s to approximately 80,964 reported in 2017 [6].

Scaling up HIV care by increasing the number of HIV treatment facilities, increasing human resources, and ensuring the availability of medical supplies and commodities has resulted in an increased number of people on ART treatment, leading to a reduction in HIV mortality [6]. Moreover, the scaling up of ART services in Tanzania has minimized the number of new infections by 13%, resulting in a decreased mortality rate of 50% between 2010 and 2018 [3]. All of these efforts combined has resulted in a steady decrease in national HIV prevalence among adults aged 15 to 49 years old, from 7% to 4.6% in the past 15 years [2,3,7,8]. Nevertheless, moving towards achieving UNAIDS 90-90-90 targets (90% of all PLHIV will know their HIV status, 90% of those diagnosed to have HIV infection will be on ART, and 90% of those on ART will achieve sustainable viral suppression by 2020) Tanzania HIV Impact Survey 2016–2017 has showed that 60.6% of PLHIV aged 15 years or older knew their HIV status. Of those who knew their status, 93.6% were on ART, and 87.0% of those on ART were virally suppressed [9].

Initially, public health facilities were the major providers of HIV services in Tanzania. However, private and faith-based health facilities were later integrated into the provision of services, resulting in several potential advantages. These included a reduced volume of patients in public facilities, a decrease in stigmatization due to the disease, shorter wait times as well as an enhanced accessibility to health services [10]. This has positively eased the burden of resources and services provided to PLHIV by the public health facilities [10,11].

Clients’ satisfaction with HIV/AIDS services plays an essential role in designing and evaluating modern health care organizations, leading to improvements in delivery systems to PLHIV [12–14]. It has been documented that clients’ satisfaction with HIV/AIDS services results in important behavioral changes that include maintaining stable relationships with health care providers, increasing compliance with therapy and treatment, and improving attendance for subsequent clinical follow up [12,15–17].

Several factors have been identified as influencing clients’ satisfaction with HIV/AIDS services. These include the availability and attitude of staff, privacy and confidentiality, facility cleanliness, and time spent in the clinic [12,16,18]. Moreover, issues related to cultural norms or religious beliefs were also found to be important factors that significantly influenced clients’ satisfaction [12]. Furthermore, several studies have reported a significant difference in clients’ satisfaction between public and private health facilities regarding time spent in the facility, privacy, cleanliness of the facilities, and staff-patient communication [17,19–21]. This divergence in satisfaction between public and non-public health facilities may be due to the organization of health services, health facility space, number of staff, drug availability, investigations during visits, disease stage, and psychological well being of participants [17].

So far, few studies have compared clients’ satisfaction with the HIV services provided at public versus private health facilities [17,21,22]. Only one study in Tanzania was found comparing clients’ satisfaction with HIV services in a public versus private delivery system, but it focused only on client’s satisfaction in laboratory services that conducted HIV-related testing [19]. Other available studies have focused only on a single type of health facility ownership [16,23,24]. Due to socio-cultural differences, it is difficult to generalize clients’ satisfaction with care and treatment centres (CTCs) based on the type of health facility ownership in other countries and compare that to Tanzania. Therefore, this study aims to assess clients’ satisfaction with different aspects of care and to determine if the type of facility ownership is a predictor of satisfaction in Ubungo District, Dar es Salaam, Tanzania.

## Materials and methods

### Study design and setting

**Fig 1:**
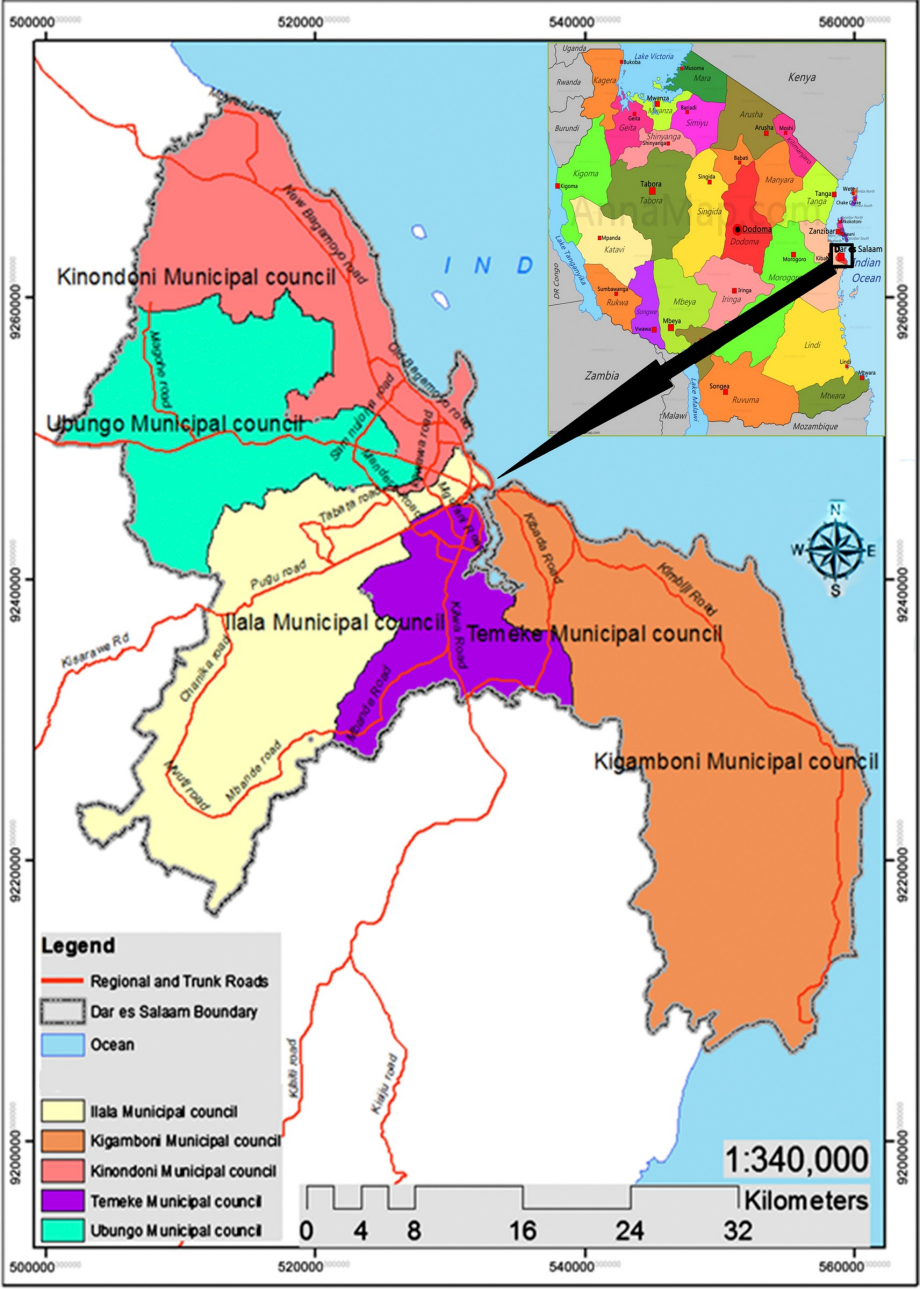
A map of Dar es Salaam, Tanzania showing Ubungo District/Municipal, the study area. Source: Google.

We conducted a cross-sectional study at Ubungo District, in Dar es Salaam Tanzania. Ubungo is one of two newly established districts in the city of Dar es Salaam, which consists of five districts. Ubungo is located in the northwest area of the city (Fig 1) and measures 10.17 km^2^, a population of 56, 016 [25]. Currently, the Ubungo district has a total of 37 health facilities providing CTC and PMTCT services; 23 dispensaries (18 public, 5 private), 6 health centres (3 public, 3 private), and 4 faith-based health facilities. Health facilities were categorized according to the type of ownership: public health centres, private health centres, and faith-based health facilities.

The health system in Tanzania operates in a referral pyramid structure, starting from the dispensary and rural health centres (RHCs) which see the greatest number of patients. These then refer to district hospitals when a higher level of care is required, after which referrals go to regional/zonal hospitals, and finally to the national and specialty hospitals which provide the highest level of care [26]. HIV/AIDS care and treatment services are integrated into all levels of health facilities; however, the package of services offered at each level differs. Lower level health facilities (such as RHCs) offer standard services which include HIV testing, prevention, and ART initiation services. At the district hospital level, the same standard array of services is provided, in addition to community outreach testing for HIV. This provides critical outreach to vulnerable populations such as adolescents, prisoners, men who have sex with men, sex workers, and people who inject drugs [27].

In Tanzania, public, private, and faith-based health facilities are the major providers of HIV services to PLHIV. In this study, a “public health facility” is defined as a provider of health services that is under government control. A “private health facility” is defined as one that is owned and controlled by an individual or organization, and a “faith-based health facility” is one that is owned and controlled by a religious institution. All of these facilities offer similar HIV services including: ART initiation/refill, clinical management, adherence support, laboratory testing, opportunistic infections treatment, and psychosocial support [5]. The provision of HIV services at these facilities is supported mainly by international donors through the government, thus providing free access to HIV services for the population except for some laboratory investigations. The laboratory tests which are provided free of charge include the CD4 count and viral load test. However, the payments for laboratory tests at public health facilities are inexpensive due to government subsidies, compared to private and faith-based facilities.

### Study population and sampling

In 2019, approximately 18,000 PLHIV were receiving CTC services in the Ubungo district. For this study, we recruited individuals who were receiving CTC services at selected health centres on the working days between September and October 2019. We included all willing respondents above 18 years old. Clients who were mentally and physically unstable were excluded. Mental stability was assessed through the researcher’s initial interaction with the respondents, and physical stability through observation and client self-report.

Ubungo district was selected purposely because it was a new area and there was little information regarding clients’ satisfaction with HIV services. The study covered over 50% of all health centres providing CTC services in the Ubungo district [28]. A list of health centres offering CTC services was obtained from the Ubungo District AIDS coordinator. We divided this list into three strata: public, private, and faith-based. From each stratum, two health facilities were chosen based on the number of clients received per day (minimum of 10 clients per day). A convenient sampling technique was used to select potential respondents for the study.

The sample size was calculated by using a formula for estimating a single population proportion for a cross-sectional study for an infinite population [29]. The expected proportion of 57.7% of patients’ satisfaction with health care services provided at the HIV clinic [30] at 95% confidence interval and 5% margin error was used. This gave a total of 417 respondents with a 10% non-response rate [31] as a calculated sample size. However, based on the respondents`availability and readiness, a total of 430 respondents attending CTC services were recruited in this study. Based on study sites pre-survey, the sample size from each type of facility was calculated depending on the maximum number of clients received per day. Fifty percent of the sample size was drawn from public health facilities and the other 50% from private and faith-based health facilities (25% each).

### Data collection and variables

Clients who were at the CTCs during the time of data collection were conveniently recruited into the study. A private consultation room, far from where care was delivered in order to ensure client anonymity, was used during data collection. In this way, study participants who provided their consent were assured of the highest level of confidentiality. With the aid of structured questions written in English and later translated into the Swahili language, we collected data through face-to-face exit interviews. The tool was adopted from an unpublished research study assessing clients’ satisfaction with family planning services in Dar es Salaam, Tanzania. This tool was modified to meet the current research objectives.

The questionnaire had four parts: Part One included facility detail (public vs. private vs. faith-based); Part Two had baseline information which included socio-demographic information (age, sex, education, marital status, and economic activity), distance to the health facility, cost of transport to and from the health facility, time enrollment to CTC and time spent in the facility; Part Three reviewed the clients’ satisfaction with different aspects of care and overall satisfaction with this care; and the last part was facility preference. In measuring clients’ satisfaction in these different aspects of care, a five point Likert-scale that ranged from very dissatisfied (1) to very satisfied (5) was used. A respondent had to select very dissatisfied (1), unsatisfied (2), uncertain (3), satisfied (4) or very satisfied (5). Also, open-ended questions to elicit the client’s reasons for dissatisfaction were included. Overall satisfaction was assessed with a "yes" or "no" question. Pre-testing of the tool was conducted among 42 clients attending a public CTC (this facility was excluded during the data collection period). All ambiguities identified during pre-testing were addressed accordingly. The reliability of the tool in question was tested and the Cronbach’s alpha was 0.71.

### Data analysis

The responses were coded, entered and run through the Statistical Package software for Social Sciences (SPSS) version 20.Data was verified for accuracy and consistency before analysis. Baseline characteristics (including socio-demographics), clients’ satisfaction with different aspects of care, and general satisfaction were described using frequencies, percentages, tabulation, graphs, mean, and median. When analyzing the results of the Likert scale to measure clients’ satisfaction with aspects of care, “very satisfied” and “satisfied” were considered to be "satisfied"; and “uncertain”, “dissatisfied”, and “very dissatisfied” were considered to be “dissatisfied". General satisfaction with care provided at CTCs was computed by finding the mean score of the six assessed aspects of care of clients’ satisfaction whereas, a mean score of ≥4 (80% and above) was regarded “satisfied” and mean score of <4 (below 80%) was regarded “dissatisfied”. Chi-square and Fisher’s Exact test were used to assess the association between baseline characteristics and clients’ satisfaction with the six assessed aspects of care. The aspects of care which showed association with the type of health facility at a p˂0.05 were examined with binary logistic regression analysis to control for confounders and obtain the direction of the relationship at a 5% level of significance.

### Ethical clearance

Ethical clearance with a Ref. No.DA.25/111/01/ was obtained from the Muhimbili University of Health and Allied Sciences Senate Research and Publication Committee. Permission to conduct a study in the selected health facilities (Ref. No.UBMC/MED/TRA/174-180) was obtained from the respective administrative authorities. All Administrators within each facility were informed about the study. Potential study respondents were informed about their rights to participate and that non-participation would not interfere with getting services at the respective facilities. Before the interview, each respondent was informed of the freedom to skip any question and to stop the interview at any time. Written consent was obtained from each respondent. For confidentiality purposes, code numbers were used to facilitate data entry, cleaning, and further processes instead of names.

## Results

### Baseline characteristics

Of the 430 respondents, 211 (49.1%) were from public health facilities, 121 (28.1%) from faith-based facilities and 98 (22.8%) were from private health facilities. The median age was 40 years, and ranged from 18 to 73 years old. The majority 279 (64.9%) were females. Almost half of the respondents, 202 (47.0%), were married, 313 (72.8%) were self-employed, and 278 (64.7%) reported that primary education was their highest level of education.

The majority of the respondents, 309 (71.8%), took less than an hour to reach the health facility, 219 (50.9%) required less than an hour to wait for services, and the majority 351 (81.6%) were enrolled in the CTC for more than a year (Table 1).

**Table 1:** Baseline characteristics of the study respondents attended CTCs in Ubungo District, Dar es Salaam between September and October 2019.

Baseline characteristics measures		Type of health facility			TOTAL (n = 430) n (%)	p-value
		Public (n = 211) n (%)	Private (n = 98) n (%)	Faith-based (n = 121) n (%)		
Age (years)	˂30	24 (11.4)	7 (7.1)	24 (19.8)	55 (12.8)	0.031*
	30–39	63 (29.9)	40 (40.8)	42 (34.7)	145 (33.7)	
	40–49	71 (33.6)	34 (34.7)	33 (27.3)	138 (32.1)	
	>49	53 (25.1)	17 (17.3)	22 (18.2)	92 (21.4)	
Sex:	Male	88(41.7)	30 (30.6)	33 (27.3)	151 (35.1)	0.017*
	Female	123(58.3)	68 (69.4)	88 (72.7)	279 (64.9)	
Marital status:	Single	74 (35.1)	18 (18.4)	20 (16.5)	112 (26.0)	˂0.001®
	Married	92 (43.6)	44 (44.9)	66 (54.5)	202 (47.0)	
	Widow/widower	33 (15.6)	36 (36.7)	32 (26.4)	101 (23.5)	
	Divorced	12 (5.7)	0 (0.0)	3 (2.5)	15 (3.5)	
Education level:	No formal education	20 (9.5)	2 (2.0)	5 (4.1)	27 (6.3)	0.001*
	Primary education	145 (68.7)	55 (56.1)	78 (64.5)	278 (64.7)	
	Secondary education	42 (19.9)	30 (30.6)	31 (25.6)	103 (24.0)	
	College education	4 (1.9)	11 (11.2)	7 (5.8)	22 (5.1)	
Occupation:	Employed	31 (14.7)	19 (19.4)	26 (21.5)	76 (17.7)	0.038*
	Self-employed	166 (78.7)	70 (71.4)	77 (63.6)	313 (72.8%)	
	Unemployed	14 (6.6)	9 (9.2)	18 (14.9)	41 (9.5)	
Enrollment with CTC services:	Less than 3 months	4 (1.9)	7 (7.1)	5 (4.1)	16 (3.7)	˂0.001®
	3–6 months	4 (1.9)	9 (9.2)	6 (5.0)	19 (4.4)	
	7–12 months	12 (5.7)	20 (20.4)	12 (9.9)	44 (10.2)	
	More than a year	191 (90.5)	62 (63.3)	98 (81.0)	351 (81.6)	
Distance (Travelling) to the health facility:	Less than 1hour	158 (74.9)	73 (74.5)	78 (64.5)	309 (71.9)	0.209®
	1–3 hours	51 (24.2)	25 (24.5)	42 (34.7)	118 (27.4)	
	More than 3 hours	2 (0.9)	0 (0.0)	1 (0.8)	3 (0.7)	
Time spent in the health facility:	Less than I hour	30 (14.2)	91 (92.9)	98 (81.0)	219 (50.9)	˂0.001*
	1–3 hours	154 (73.0)	7 (7.1)	21 (17.4)	182 (42.3)	
	More than 3 hours	27 (12.8)	0 (0.0)	2 (1.7)	29 (6.7)	

*p-value of Chi-square;

®p-value of Fisher’s test; n = Frequency.

### Clients’ satisfaction with CTC services

The general clients’ satisfaction from all health facilities was 92.3%.Out of the total number of respondents, 95.7% were from public facilities, 93.9% from private facilities, and 85.1% from faith-based facilities. This difference was found to be statistically significant with a p-value of 0.002.

Clients’ satisfaction with six aspects of care was assessed: these were (1) time spent in the facility, (2) privacy and confidentiality, (3) physical environment, (4) counseling, (5) staff-patient communication, and (6) ART availability. Regardless of the type of health facility (public vs. private vs. faith-based), respondents reported being most satisfied with ART availability (98.1%) and most dissatisfied with time spent at the health facility (14.7%) while receiving services (Table 2). Those respondents dissatisfied with CTC services mostly complained of the long wait time and the physical environment at the respective facilities.

**Table 2:** The association between baseline characteristics and respondents’ satisfaction with aspects of care in Ubungo District, Dar es Salaam between September and October 2019.

Variables	Time spent in the facility		Privacy and confidentiality		Physical environment		Counseling		Staff-patient communication		ART availability	
	Satisfied n (%)	p-value	Satisfied n (%)	p-value	Satisfied n (%)	p-value	Satisfied n (%)	p-value	Satisfied n (%)	p-value	Satisfied n (%)	p-value
**Facility type**												
Public	168 (79.6)	0.001*	211 (100)	**<0.001®**	211 (100)	**<0.001®**	210 (99.5)	**0.037®**	197 (93.4)	**0.031®**	210 (99.5)	0.056®
Private	94 (95.9)		92 (93.9)		96 (98)		94 (95.9)		95 (96.9)		95 (96.9)	
Faith-based	105 (86.8)		110 (90.9)		104 (86)		117 (96.7)		120 (99.2)		117 (96.7)	
**Age (years)**												
Less than 35 years	94 (81)	0.127*	109 (94)	0.262®	105 (90.5)	0.004*	110 (94.8)	**0.014**®	107 (92.2)	**0.032®**	113 (97.4)	0.450®
35 years or older	273 (86.9)		304 (96.8)		306 (97.5)		311 (99)		305 (97.1)		309 (98.4)	
**Sex**												
Male	134 (88.7)	0.155*	149 (98.7)	0.066*	150 (99.3)	0.005*	150 (96.3)	0.170®	144 (95.4)	0.802*	147 (97.4)	0.460®
Female	233 (83.5)		264 (94.6)		261 (93.5)		271 (77.1)		268 (96.1)		275 (98.6)	
**Marital status**												
Single	199 (87.3)	0.274*	221 (96.9)	0.334*	215 (94.3)	0.240*	224 (98.2)	0.740®	222 (97.4)	0.097*	224 (98.2)	1.000®
Married	168 (83.2)		192 (95)		196 (97)		197 (97.5)		190 (94.1)		198 (98)	
**Education**												
Primary or less	261 (85.6)	0.881*	294 (96.4)	0.589®	298 (97.7)	0.002*	299 (98)	0.723®	291 (95.4)	0.606*	302 (99)	**0.049®**
Secondary or more	106 (84.8)		219 (95.2)		113 (90.4)		122 (97.6)		121 (96.8)		120 (96)	
**Occupation**												
Employed	333 (85.6)	0.817*	376 (96.7)	0.068®	373 (95.9)	0.410®	381 (97.9)	0.598®	375 (97.4)	0.081®	382 (98.2)	0.555®
Unemployed	34 (82.9)		37 (90.2)		38 (92.7)		40 (97.6)		37 (90.2)		40 (97.5)	
**CTC enrollment**												
1 year or less	69 (87.3)	0.606*	72 (91.1)	**0.022®**	76 (96.2)	1.000®	75 (94.9)	0.064®	75 (94.9)	0.755®	78 (98.7)	1.000®
More than a year	298 (84.9)		341 (97.2)		335 (95.4)		346 (98.6)		337 (96)		344 (98)	
**Time spent in the facility**												
Less than 1 hour	212 (96.8)	<0.001*	208 (95)	0.324*	203 (92.7)	0.004*	215 (98.2)	0.747®	215 (98.2)	0.015*	215 (98.2)	1.000®
1hour or more	155 (73.5)		205 (97.2)		208 (98.6)		206 (97.6)		197 (93.4)		207 (98.1)	

*p-value of Chi-square test;

®p-value of Fisher’s test; n = Frequency.

### Satisfaction with aspects of care according to the type of health facility

**Fig 2:**
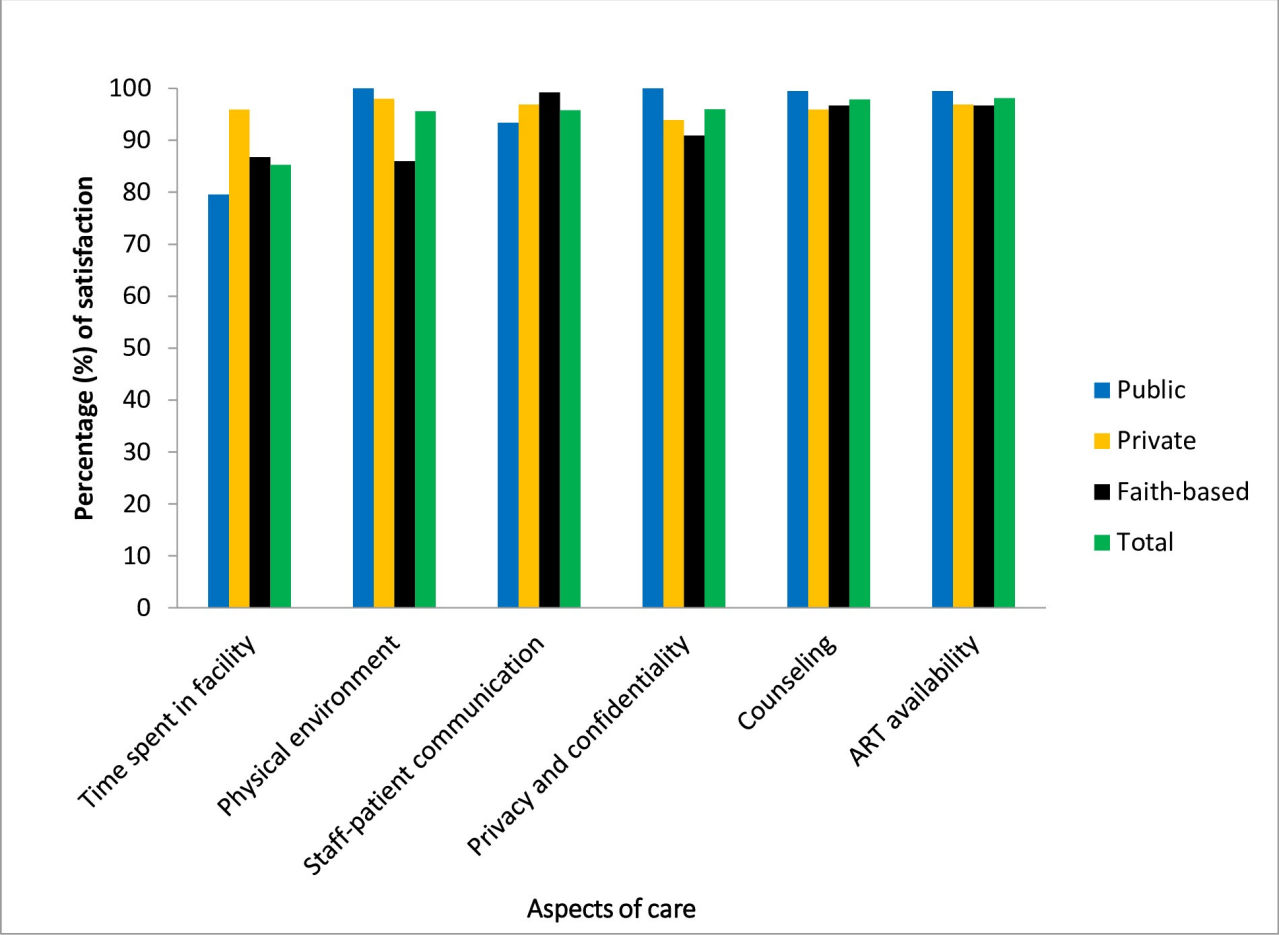
Satisfaction with aspects of care according to the type of health facility ownership in Ubungo District, Dar es Salaam between September and October 2019.

Respondents from public health facilities were most satisfied with privacy and confidentiality (100%), physical environment (100%), counseling (99.5%) and drug availability (99.5%) whereas those from private health facilities were most satisfied with the time spent in the facility (95.9%). Finally, respondents from faith-based health facilities were most satisfied with staff-patient communication (99.2%) (Fig 2). The association between type of health facility and all assessed aspects of care was found to be statistically significant except for ART availability (Table 2).

In univariate binary logistic regression analysis, satisfaction with two aspects of care (time spent in the facility, and staff-patient communication) showed significant association with the type of health facility. The odds of being satisfied with the time spent in the facility for respondents in private health facilities were 6 times more than for those who attended public health facilities (95% CI: 2.094–17.278). The odds of being satisfied with staff-patient communication for respondents from faith-based health facilities were 8.5 times more than for those who attended public health facilities (95% CI: 1.107–65.677) (Table 3).

**Table 3:** Univariate binary logistic regression of association between type of health facility ownership and satisfaction with aspects of care in Ubungo District, Dar es Salaam between September and October 2019.

Variables	Time spent in the facility		Counseling		Staff-patient communication	
	COR (95% CI)	p-value	COR (95% CI)	p-value	COR (95% CI)	p-value
**Type of health facility**						
Public	1		1		1	
Private	6.01 (2.09–17.28)	**0.001**	0.11 (0.01–1.03)	0.052	2.25 (0.63–8.02)	0.211
Faith-based	1.68 (0.90–3.13)	0.103	0.14 (0.02–1.26)	0.079	8.53 (1.12–65.68)	**0.040**

COR = Crude Odds Ratio; CI = Confidence Interval.

After adjusting for other baseline characteristics, the type of health facility remained significantly associated with the aspect of care known as “satisfaction with time spent in the facility”. Respondents who spent more than one hour in the faith-based health facilities had lower odds of being satisfied with the time spent there compared to those who spent more than one hour in public health facilities (OR = 0.31, 95% CI: 0.14–0.71, p = 0.006) (Table 4).

**Table 4:** Multiple logistic regression of three satisfaction aspects of care on type of facility, adjusting for other variables in Ubungo District, Dar es Salaam between September and October 2019.

Variables	Time spent in the facility		Counseling		Staff-patient communication	
	AOR (95% CI)	p-value	AOR (95% CI)	p-value	AOR (95% CI)	p-value
**Type of health facility:**						
Public	1		1		1	
Private	0.72 (0.21–2.54)	0.614	0.11 (0.01–1.03)	0.053	1.13 (0.20–6.36)	0.893
Faith-based	0.31 (0.14–0.71)	**0.006**	0.19 (0.02–1.72)	0.141	6,27(0.67–58.50)	0.107
**Age (years)**						
Less than 35 years			0.19 (0.05–0.81)	**0.024**	0.26 (0.10–0.69)	**0.007**
35 years or older			1		1	
**Time spent in the facility**						
Less than 1 hour	1				1	
1hour or more	0.05 (0.02–0.14)	**<0.001**			0.37 (0.08–1.80)	0.219

AOR = Adjusted Odds Ratio; CI = Confidence Interval.

## Discussion

The primary purpose of this study was to assess clients’ satisfaction with different aspects of care and to determine if the type of facility ownership was a predictor of satisfaction with these aspects of care. Clients’ satisfaction with HV/AIDS services is an important outcome of the delivery of healthcare to this particular population as it can affect the compliance with care and thereby impact the health outcome of PLHIV. In general, clients’ satisfaction with CTC services in the current study was high. Also, the type of health facility ownership was found to be associated with increased satisfaction with time spent in the facility.

In this study, clients’ satisfaction with HIV services provided at CTCs in the Ubungo district was high. This may be due to increased access to care and treatment and quality of health services, particularly in urban areas. A high level of satisfaction in the current study is consistent with other studies that were conducted in Dar es Salaam-Tanzania, Bamenda-Cameroon, and Nigeria [13,16,23,32]. This is in direct contrast with studies that were done in Vietnam, Uganda, and Pakistan which reported low overall satisfaction with CTC services [14,22,30]. This difference could be contributed to differences in respondents’ characteristics, the use of a new tool in a previous study [22], and the study being done in a resource-scarce setting [14].

“Time spent” is the duration of time that a client spends at the facility from arrival to completion of the visit. Most of the respondents reported being satisfied with the time spent in the facility when they were able to spend less than one hour. In the present study, satisfaction with this variable might be due to having a larger number of staff available, or because of a higher work ethic in the staff, or because respondents went for fewer services (such as drug refills) and not to receive comprehensive services that might include laboratory investigations which are usually time-consuming. This result is similar to the results from a study conducted in Nigeria in which respondents were highly satisfied with the wait time [32]. In our research, the percentage of respondents “satisfied with the time spent in the facility” was high compared to studies done in KwaZulu-Natal and Nigeria in which only half of the respondents reported being satisfied with wait time [12,33]. In addition, in a study done in Vietnam, more than half of the respondents were dissatisfied with wait time [14]. However, none of these studies specified the exact time that clients spent at the CTCs. In our study, most dissatisfied respondents stated that the wait time before services were provided was a reason for dissatisfaction. This is because most of them arrive early in the morning before working hours so they can be served first to avoid the long queue ("first come, first served"). Unfortunately, many clients came at this early time, leading to the longer wait times.

Privacy is a crucial aspect of care that influences satisfaction with HIV/AIDS services among PLHIV. It enables them to conceal their status from other people and thereby avoid stigmatization from society. In addition, privacy provides a more comfortable environment for clients, allowing for closer engagement with centre staff, and facilitating better communication [16,34]. Findings from our study reveal that the majority of respondents were satisfied with privacy and confidentiality. The introduction and expansion of HIV training programs may substantially influence health professionals to be conscious in adhering to profession-related ethics. Similarly, a previous study in Cameroon showed that respondents were satisfied with privacy and confidentiality [13]. Compared to a similar study in Vietnam, where 60.1% of respondents were satisfied with privacy and confidentiality in service delivery [14], the level of client satisfaction with privacy in the present study was extremely high.

Effective communication in health care settings improves compliance with treatment, their outcomes, and the overall quality of care [35–37]. This study found high satisfaction with staff-patient communication. This might be due to the absence of stigma to PLHIV among health care providers and their willingness in dedicate time to listen to clients’ concerns. Studies conducted in Nigeria support our finding of high satisfaction with staff-patient communication [15,17,37]. However, in studies done in Cameroon and KwaZulu-Natal, most respondents were dissatisfied with staff-patient communication due to the language barrier and the fact that health workers were too busy to listen to patients’ problems [13,36].

Counseling is essential among PLHIV because of the challenges that this group of people encounters. These can include stigmatization, the difficulty of developing coping strategies in order to maintain both physical and emotional health, as well as the importance of reducing high risk behavior such as unsafe sexual practices [38]. The majority of respondents were highly satisfied with the counseling received at CTCs. Health education is given to PLHIV in every visit. This includes counseling and support around the importance of adhering to treatment, eating a balanced diet, doing regular exercise, as well as advice about possible drug side effects. This is consistent with a study done in Dar es Salaam, Tanzania which revealed the majority of respondents were satisfied with counseling given at CTCs [23].

Although it has been reported that clients’ satisfaction with care was centred more on the process of receiving care than on infrastructure, the latter has also been associated with clients’ satisfaction [23,37,39]. In our study, the majority of respondents reported being highly satisfied with the facilities’ physical environment: its cleanliness, organization, and overall attractiveness. This finding was consistent with that of a study done in Dar es Salaam where all respondents were highly satisfied with the physical environment [16]. However, this result was dissimilar to previous studies from another municipality in Dar es Salaam and KwaZulu-Natal which showed dissatisfaction with the facility’s physical environment. The reasons for this dissatisfaction included water availability, cleanness, and the location of the toilets [30,36]. In the present study, dissatisfied respondents reported that the facility was too open, and thus it was too easy for them to be seen by friends, relatives, or neighbors when accessing services.

Drug availability is a crucial concern to PLHIV because it is a key element in the prevention and management of HIV drug resistance [22]. In our study, one of the aspects of care ranked highest in satisfaction was drug availability. The reason for this higher satisfaction may be explained by the free supply of ARTs from 2004 to the present. This is similar to studies done in Nigeria and Ethiopia where respondents were highly satisfied with free and available ART [15,32]. However, it contrasts with studies conducted in Uganda and Nigeria in which the respondents report drug availability as a problem [22].

After adjusting for potential confounders to determine if a type of health facility was a predictor of clients’ satisfaction with aspects of care, we found that the type of health facility was significantly associated with only one aspect of care: “time spent in the facility”. Respondents who spent more than one hour in the faith-based health facilities were less likely to be satisfied compared to those who spent more than one hour in public health facilities. This may be explained by the fact that people with low incomes seek health services in public health facilities given their lower cost. This may result in clients having lower expectations from the system, including the wait time they spend at the facility receiving services. This finding was corroborated by a study done in Nigeria which showed an association between the type of health facility ownership and satisfaction with time spent with the doctor [17].

The present study has several limitations. First, it was not possible to assess an individual’s satisfaction over time because this was a cross-sectional study. However, it was useful in assessing the association between the type of health facility and satisfaction with different aspects of care. Second, due to a limited number of potential respondents in the selected settings, respondents at the health facilities were selected conveniently making the sample not representative of the population of PLHIV and therefore limiting the generalization of the study findings. Nonetheless, this study was a multi-centre study and the recruited sample was adequate to highlight the context-specific general satisfaction with HIV/AIDS at CTCs. Third, despite the tool being reliable, the questionnaire was not configured to handle social desirability bias because it consisted of only positively constructed questions. Therefore, this may explain the attained high satisfaction level in this study. Lastly, respondents might have withheld information about their negative experiences and instead expressed satisfaction, despite the fact that respondents had been assured a high level of confidentiality and their privacy was well maintained.

## Conclusion

Generally, clients’ satisfaction with HIV/AIDS services at CTCs in the Ubungo District, Dar es Salaam was high. Also, the type of health facility was found to be a predictor of satisfaction with one aspect of care: that is, time spent in the facility. The former finding should encourage health care providers to maintain high-quality services to sustain clients’ satisfaction. Additionally, assessment of clients’ satisfaction with HIV/AIDS services should be carried out regularly for better health outcomes of PLHIV.

## Supporting information

S1 File English questionnaire. (PDF)

S2 File Swahili questionnaire. (PDF)

## References

[1] World Health Organization. HIV/AIDS [Internet]. 2019 [cited 2020 Feb 29]. Available from: http://www.who.int/news-room/fact-sheets/detail/hiv-aids.

[2] Tanzania Commission for AIDS (TACAIDS) National Bureau of Statistics (NBS), Office of the Chief Government Statistician (OCGS), ICF International ZAC (ZAC). Tanzania HIV/AIDS and malaria indicator survey 2011–12. Co-published by ZAC, NBS, OCGS, and ICF International. TACAIDS Dar es Salaam (Tanzania); 2013.

[3] Avert. HIV and AIDS in Tanzania: Global information and education on HIV and AIDS [Internet]. 2019 [cited 2020 Feb 29]. Available from: https://www.avert.org/professionals/hiv-around-world/sub-saharan-africa/tanzania.

[4] Ministry of Health and Social Welfare; National AIDS Control Programme. Implementation of HIV/AIDS Care and Treatment Services in Tanzania. 2011.

[5] National AIDS Control Programme. National Guidelines for the Management of HIV and AIDS. Sixth Edit. Ministry of Health, Community Development, Gender, Elderly and Children. National AIDS Control Programme; 2017.

[6] Tanzania Commission for AIDS (TACAIDS). National HIV and AIDS Response Report for 2017. 2017.

[7] Joint United Nations Programme on HIV/AIDS (UNAIDS). UNAIDS Data 2017. UNAIDS Joint United Nations Programme on HIV/AIDS 2017. doi:978-92-9173-945-5. 12349391

[8] National bureau of Statistics (NBS) and ORC Marco. Tanzania Demographic and Health Survey 2004–05. National Bureau of Statistics and ORC Macro. Dar es Salaam: Tanzania; 2005. doi:zania Demographic and Health Survey 2004–05. Dar es Salaam, Tanzania: National Bureau of Statistics and ORC Macro.

[9] Tanzania Commission for AIDS (TACAIDS) International; Zanzibar AIDS Commission (ZAC). Tanzania HIV Impact Survey (A Population-Based HIV Impact Assessment) THIS 2016–2017. 2018.

[10] Sargent J, Johnson J, Majorowski M, Friedman N, Blazer C. Private sector involvement in HIV service provision. Arlington, VA: USAID AIDS Support and Technical Assistance Resources, AIDSTAR-One Project, Task Order 1. 2009.

[11] PEPFAR. PEPFAR 2015 Annual Report to Congress [Internet]. U.S. President’s Emergency Plan for AIDS Relief. 2015. Available from: https://www.state.gov/wp-content/uploads/2019/08/PEPFAR-2015-Annual-Report-to-Congress.pdf.

[12] Anosike A, Olakunde BO, Adeyinka DA, Ezeokafor C, Amanze O, Mathews O, et al Clients’ satisfaction with HIV treatment and care services in Nigeria. Public Health. 2019;167:50–4. 10.1016/j.puhe.2018.11.012 30639803

[13] Wung BA, Peter NF, Atashili J. Clients’ satisfaction with HIV treatment services in Bamenda, Cameroon: A cross-sectional study. BMC Health Serv Res. 2016;16(1):1–9. 10.1186/s12913-016-1512-5 27431998 PMC4950718

[14] Tran BX, Nguyen NPT. Patient Satisfaction with HIV/AIDS Care and Treatment in the Decentralization of Services Delivery in Vietnam. Cameron DW, editor. PLoS One. 2012 10 5;7(10):e46680 10.1371/journal.pone.0046680 23071611 PMC3465274

[15] Abebe TB, Erku DA, Gebresillassie BM, Haile KT, Mekuria AB. Expectation and satisfaction of HIV/AIDS patients toward the pharmaceutical care provided at Gondar university referral hospital, northwestern Ethiopia: A cross-sectional study. Patient Prefer Adherence. 2016;10:2073–82. 10.2147/PPA.S114720 27784997 PMC5063358

[16] Kagashe GAB, Rwebangila F. Patient satisfaction with health care services provided at HIV clinics at Amana and Muhimbili hospitals in Dar Es Salaam. Afr Health Sci. 2011;11(1). 10.4314/ahs.v11i3.70072 22135647 PMC3220117

[17] Osungbade KO, Shaahu VN, Owoaje EE, Adedokun BO. Patients’ Satisfaction with Quality of Anti-Retroviral Services in Central Nigeria: Implications for Strengthening Private Health Services. World J Prev Med World J Prev Med. 2013;1(3):11–8. 10.12691/jpm-1-3-1

[18] Devnani M, Gupta AK, Wanchu A, Sharma RK. Factors associated with health service satisfaction among people living with HIV/AIDS: A cross sectional study at ART center in Chandigarh, India. AIDS Care—Psychol Socio-Medical Asp AIDS/HIV. 2012;24(1):100–7. 10.1080/09540121.2011.592816 21767229

[19] Mfinanga SG, Kahwa A, Kimaro G, Kilale A, Kivuyo S, Senkoro M, et al Patient’s dissatisfaction with the public and private laboratory services in conducting HIV related testing in Tanzania. BMC Health Serv Res. 2008;8:1–5. 10.1186/1472-6963-8-1 18687113 PMC2519070

[20] Shet A, Decosta A, Heylen E, Shastri S, Chandy S, Ekstrand M. High rates of adherence and treatment success in a public and public-private HIV clinic in India: Potential benefits of standardized national care delivery systems. BMC Health Serv Res. 2011;11:1–8. 10.1186/1472-6963-11-1 22004573 PMC3204232

[21] Umeokonkwo CD, Aniebue PN, Onoka CA, Agu AP, Sufiyan MB, Ogbonnaya L. Patients’ satisfaction with HIV and AIDS care in Anambra State, Nigeria. PLoS One. 2018;13(10):1–14. 10.1371/journal.pone.0206499 30365560 PMC6203402

[22] Kwesiga D, Kiwanuka S, Kiwanuka N, Mafigiri D, Nelson K. The clients’ Voice: Satisfaction with HIV/AIDS care in a public and private health facility in Kabale District, Uganda. J AIDS Clin Res. 2013;4(7). 10.4172/2155-6113.1000220

[23] Ndayongeje J, Kazaura M. Satisfaction of patients attending public HIV or AIDS care and treatment centers in Kinondoni district, Tanzania. Int Q Community Health Educ. 2017;37(2):113–9. 10.1177/0272684X17701264 28511601

[24] Miller JS, Mhalu A, Chalamilla G, Siril H, Kaaya S, Tito J, et al Patient satisfaction with HIV/AIDS care at private clinics in Dar es Salaam, Tanzania. AIDS Care. 2014;26(9):1150–4. 10.1080/09540121.2014.882487 24499337 PMC4465080

[25] United Republic of Tanzania; National Bureau of Statistics. The 2012 Population and Housing Census: Population Distribution by Administrative Areas. National Bureaul of Statistics Dar es Salaam; 2013.

[26] Mselle LT, Aston M, Kohi TW, Mbekenga C, Macdonald D, White M, et al The Challenges of Providing Postpartum Education in Dar es Salaam, Tanzania: Narratives of Nurse-Midwives and Obstetricians. Qual Health Res. 2017;27(12):1792–803. 10.1177/1049732317717695 28705071

[27] National AIDS Control Programme (NACP). Operational Manual for Comprehensive Differentiated Delivery of HIV and AIDS Services. Ministry of Health, Community Development, Gender, Elderly and Children, National AIDS Control Programme; 2019.

[28] Kielmann A, Janovsky K, Annett K. Protocol for rapid data collection and analysis Assessing District Health Needs, Services and Systems. London: Macmillan Education LTD; 1995.

[29] Kirkwood BR, Sterne JAC. Essential medical statistics. John Wiley & Sons; 2010.

[30] Bhutto A-Q, Nisar N. Health-seeking behaviour of people living with HIV/AIDS and their satisfaction with health services provided at a tertiary care hospital, Karachi, Pakistan. East Mediterr Heal J. 2017;23(1):13–9. 10.26719/2017.23.1.13 28244056

[31] Israel GD. Determination of sample size. Malaysian J Med Sci. 2003;10(2):84–6. PMC3561892 23386802

[32] Oche MO, Raji MO, Kaoje AU, Gana G, Ango JT, Okafoagu N, et al Clients satisfaction with anti retroviral therapy services in a tertiary hospital in Sokoto, Nigeria. J AIDS HIV Res. 2013;5(9):328–33. 10.5897/JAHR2013.0247

[33] Azuike E, Adinma E, Umeh U, Njelita I, Anemeje O, Aniemena R. Client’s Satisfaction with Waiting Time in HIV Treatment Centers: An Urban Rural Comparison in Anambra State, Nigeria. Epidemiol Open Access. 2017;07(01). 10.4172/2161-1165.1000289

[34] Dapaah JM, Senah KA. HIV/AIDS clients, privacy and confidentiality; The case of two health centres in the Ashanti Region of Ghana. BMC Med Ethics [Internet]. 2016;17(1):1–10. Available from: 10.1186/s12910-016-0123-3 27422295 PMC4947355

[35] Pérez-Salgado D, Compean-Dardón MS, Staines-Orozco MG, Ortiz-Hernández L. Satisfaction with Healthcare Services and Adherence to Antiretroviral Therapy among Patients with HIV Attending Two Public Institutions. Rev Invest Clin. 2015;67(2):80–8. 25938840

[36] Chimbindi N, Bärnighausen T, Newell ML. Patient satisfaction with HIV and TB treatment in a public programme in rural KwaZulu-Natal: Evidence from patient-exit interviews. BMC Health Serv Res. 2014;14 10.1186/1472-6963-14-32 24450409 PMC3904687

[37] Adekanye AO, Adefemi SA, Okuku AG, Onawola KA, Adeleke IT, James JA. Patients’ satisfaction with the healthcare services at a north central Nigerian tertiary hospital. Niger J Med. 2013;22(3):218–24. 10.4314/njm.v22i3 24180151

[38] Centers for Disease Control and Prevention. Sexually Transmitted Diseases Treatment Guidelines [Internet]. 2015 [cited 2020 Jan 9]. Available from: https://www.cdc.gov/std/tg2015/hiv.htm. 10.1093/cid/civ771 26602614

[39] Azuike EC, Kadiri-Eneh NP, Onyemachi PE, Nwachukwu AC, Chikezie JA, Enukeme JU. Clients’ satisfaction with services in HIV treatment centres: Comparison of urban and rural centres in Anambra State, Nigeria. Int J Adv Med Sci Biotechnol. 2017;3(1).

