# Supplementary material for: Clients’ satisfaction with HIV care and treatment centres in Dar es Salaam, Tanzania: A cross-sectional study
Source: PLoS One. 2021 Feb 22;16(2):e0247421. doi: 10.1371/journal.pone.0247421 (PMC7899352; doi:10.1371/journal.pone.0247421)
Supplement: S1 File — (PDF) [file pone.0247421.s001.pdf]

ENGLISH QUESTIONNAIRE

CLIENTS' SATISFACTION WITH HIV/AIDS SERVICES AT CARE AND TREATMENT CLINICS IN UBUNGO DISTRICT, DAR ES SALAAM 2019.

Questionnaire No [ ] **Date** [ ]

PART I: FACILITY DETAIL

1. Type of health facility: Private/ Public/Faith-based

PART II: SOCIO-DEMOGRAPHIC (Put a tick (√) in a correct answer).

2. Age………..
3. Sex: Male ( ) Female ( )
4. Residency…………..
5. What is your marital status?
   a) Single ( )
   b) Married ( )
   c) Widow/widower ( )
   d) Divorced ( )
6. What is your highest level of education?
   a) No formal education ( )
   b) Primary education ( )
   c) Secondary education ( )
   d) College education ( )
7. What is your occupation?
   a) Employed ( )
   b) Self employed ( )
   c) Unemployed ( )
   d) Student ( )
8. When were you enrolled in this HIV/AIDS CTC?
   a) Less than 3 months ( )
   b) 3 – 6 months ( )

c) 7 – 12 months ( )

d) More than a year ( )

9. What is the distance of your home from the health facility?

a) Less than 1hour ( )

b) 1 – 3 hours ( )

c) More than 3 hours ( )

10. What is the cost of transport to the health facility and back to home in total?

a) I don’t use any cost ( )

b) 500 - 2000 Tshs. ( )

c) 2100 - 3500 Tshs. ( )

d) 3600 - 6000 Tshs. ( )

e) More than 6000 Tshs. ( )

11. How long have you spent in a facility?

a) Less than I hour ( )

b) 1-3 hours ( )

c) More than 3 hours ( )

PART III: RATING SCALE INVOLVING OVERALL CLIENTS’ SATISFACTION

12. (a) I would like to know your level of satisfaction in the following areas:

	Very dissatisfied	Dissatisfied	Uncertain	Satisfied	Very satisfied
Time spent in the facility					
Counseling					
Privacy and confidentiality					
Staff -Patient communication					
Physical environment					
ART availability					

(b) If not satisfied with any of the above, what is/are the reasons?

1. Time spent in the facility

……………………………………………...

……………………………………………………………………………………………….

………………………………………………………………………………………………..

1. Counseling

…………………………………………………………………………………………………

…………………………………………………………………………………………………

……………………………………………………………………………………………

2. Privacy and confidentiality

…………………………………………………………………………………………………

…………………………………………………………………………………………………

……………………………………………………………………………………………

3. Staff -Patient communication

…………………………………………………………………………………………………

…………………………………………………………………………………………………

……………………………………………………………………………………………

4. Physical environment

…………………………………………………………………………………………………

…………………………………………………………………………………………………

……………………………………………………………………………………………

5. Others

…………………………………………………………………………………………………

…………………………………………………………………………………………………

……………………………………………………………………………………………

13. Are you satisfied with the overall quality of care you receive at this facility?

a) Yes ( )

b) No ( )

PART IV: FACILITY PREFERENCE

14. I would like to know your preference of this facility:

	Very unlikely	Unlikely	Uncertain	Likely	Very likely
Likelihood of returning to this facility in the next visit					
Recommending the clinic to friends, family members and relatives					

THANKS FOR YOUR PARTICIPATION
