# Supplementary material for: Clients’ satisfaction with HIV care and treatment centres in Dar es Salaam, Tanzania: A cross-sectional study
Source: PLoS One. 2021 Feb 22;16(2):e0247421. doi: 10.1371/journal.pone.0247421 (PMC7899352; doi:10.1371/journal.pone.0247421)
Supplement: S2 File — (PDF) [file pone.0247421.s002.pdf]

DODOSO LA KISWAHILI

URIDHIKAJI WA WAGONJWA NA HUDUMA ZA UKIMWI ZINAZOTOLEWA KATIKA VITUO MBALIMBALI VYA AFYA KATIKA WILAYA YA UBUNGO, DAR ES SALAAM 2019.

Namba ya dodoso [ ] **Tarehe** [ ]

SEHEMU YA KWANZA: TAARIFA YA KITUO

1. Aina ya kituo: Binafsi/ Umma/Dini

SEHEMU YA PILI: TAARIFA BINAFSI (Weka alama ya √ sehemu inayohusika)

2. Una umri gan?.....................
3. Jinsia: Me ( ) Ke ( )
4. Mahali unapoishi………………………..
5. Hali yako ya ndoa ni ipi?
   a) Sijaoa/ Sijaolewa ( )
   b) Nimeoa/ Nimeolewa ( )
   c) Mjane/Mgane ( )
   d) Nimeachika ( )
6. Kiwango chako cha juu cha elimu ni kipi?
   a) Sijaenda shule ( )
   b) Shule ya msingi ( )
   c) Shule ya sekondari ( )
   d) Elimu ya chuo ( )
7. Unajishughulisha na nini?
   a) Nimeajiriwa ( )
   b) Nimejiajiri ( )
   c) Sina kazi ( )
   d) Mwanafunzi ( )
8. Lini ulianza kupata huduma katika kituo hiki?
   a) Chini ya miezi mitatu ( )

b) Miezi mitatu hadi sita ( )

c) Miezi saba hadi kumi na mbili ( )

d) Zaidi ya mwaka mmoja ( )

9. Kutokea unaposhi, hutumia muda gani kufika kituoni hapa?

a) Chini ya saa moja ( )

b) Saa 1 – 3 ( )

c) Zaidi ya saa 3 ( )

10. Hutumia gharama ya usafiri kiasi gani kufika kituoni hapa na kurudi nyumbani kwa ujumla?

a) Situmii gharama yoyote ( )

b) 500 - 2000 Tshs. ( )

c) 2100 - 3500 Tshs. ( )

d) 3600 - 6000 Tshs. ( )

e) Zaidi ya 6000 Tshs. ( )

11. Ni muda gani umetumia kituoni hapa toka ulipofika?

a) Chini ya saa moja ( )

b) Saa 1-3 ( )

c) Zaidi ya masaa 3 ( )

SEHEMU YA TATU: HALI YA KURIDHIKA MTEJA (Weka alama ya √ sehemu inayo/zinazohusika)

12. a) Ningependa kujua ni jinsi gani ulivyoridhika katika maeneo yafuatayo:

	Sijaridhika kabisa	Sijaridhika	Sina uhakika	Nimeridhika	Nimeridhika kabisa
Muda uliotumia kliniki					
Ushauri nasaha ulopewa					
Faragha ya huduma na Usiri wa taarifa					
Mawasiliano ya					

mtoa huduma na mgonjwa					
Mazingira ya kituo					
Upatikanaji wa dawa					

(b) Kama hujaridhishwa na huduma yeyote hapo juu, sababu ni ipi?

1. Muda uliotumia kliniki

……………………………………………………………………………………………………

……………………………………………………………………………………………………

………………………………………………………………………………………

2. Ushauri nasaha ulopewa

……………………………………………………………………………………………………

……………………………………………………………………………………………………

………………………………………………………………………………………

3. Faragha ya huduma na Usiri wa taarifa

……………………………………………………………………………………………………

……………………………………………………………………………………………………

………………………………………………………………………………………

4. Mawasiliano ya mtoa huduma na mgonjwa

……………………………………………………………………………………………………

……………………………………………………………………………………………………

………………………………………………………………………………………

5. Mazingira ya kituo

……………………………………………………………………………………………………

……………………………………………………………………………………………………

………………………………………………………………………………………

6. Upatikanaji wa dawa

……………………………………………………………………………………………………

……………………………………………………………………………………………………

………………………………………………………………………………………

7. Sababu nyinginezo zinazopelekea kutokuridhishwa na huduma katika kituo hiki

…………………………………………………………………………………………

…………………………………………………………………………………………

………………………………………………………………………………………

13. Je, unaridhishwa na huduma inayotolewa katika kituo hiki kwa ujumla?

a) Ndio ( )

b) Hapana ( )

SEHEMU YA NNE: UPENDELEO WA KITUO

14. Ningependa kufahamu upendeleo wako wa kituo hiki

	Hakuna uwezekano kabisa	Hakuna uwezekano	Sina uhakika	Kuna uwezekano	Kuna uwezekano mkubwa
Uwezekano wa kurudi tena kupata huduma katika kituo hiki					
Uwezekano wakukipendekeza kituo kwa ndugu, jamaa na marafiki					

AHSANTE KWA KUSHIRIKI
